# Sox4 represses host innate immunity to facilitate pathogen infection by hijacking the TLR signaling networks

**DOI:** 10.1080/21505594.2021.1882775

**Published:** 2021-02-16

**Authors:** Jian Shang, Yuan Zheng, Jiayin Mo, Wenbiao Wang, Zhen Luo, Yongkui Li, Xulin Chen, Qiwei Zhang, Kailang Wu, Weiyong Liu, Jianguo Wu

**Affiliations:** a Guangdong Provincial Key Laboratory of Virology,Institute of Medical Microbiology, Jinan University, Guangzhou, China; b State Key Laboratory of Virology,College of Life Sciences, Wuhan University, Wuhan, China

**Keywords:** Enterovirus 71, EV71, IFN regulatory factors 3/7, IRF3/7, hepatitis C virus, HCV, IFN-stimulated gene, ISG, influenza A virus, IAV, interferon, IFN, myeloid differentiation primary response gene 88, MyD88, sendai virus, SEV, sex-determining region Y-box 4, Sox4, Toll-like receptors, TLRS, vesicular stomatitis virus, VSV

## Abstract

Toll-like receptors (TLRs) are essential for the protection of the host from pathogen infections by initiating the integration of contextual cues to regulate inflammation and immunity. However, without tightly controlled immune responses, the host will be subjected to detrimental outcomes. Therefore, it is important to balance the positive and negative regulations of TLRs to eliminate pathogen infection, yet avert harmful immunological consequences. This study revealed a distinct mechanism underlying the regulation of the TLR network. The expression of sex-determining region Y-box 4 (Sox4) is induced by virus infection in viral infected patients and cultured cells, which subsequently represses the TLR signaling network to facilitate viral replication at multiple levels by a distinct mechanism. Briefly, Sox4 inhibits the production of myeloid differentiation primary response gene 88 (MyD88) and most of the TLRs by binding to their promoters to attenuate gene transcription. In addition, Sox4 blocks the activities of the TLR/MyD88/IRAK4/TAK1 and TLR/TRIF/TRAF3/TBK1 pathways by repressing their key components. Moreover, Sox4 represses the activation of the nuclear factor kappa-B (NF-κB) through interacting with IKKα/α, and attenuates NF-kB and IFN regulatory factors 3/7 (IRF3/7) abundances by promoting protein degradation. All these contributed to the down-regulation of interferons (IFNs) and IFN-stimulated gene (ISG) expression, leading to facilitate the viral replications. Therefore, we reveal a distinct mechanism by which viral pathogens evade host innate immunity and discover a key regulator in host defense.

## Introduction

Pathogeneses caused by microbial infections are the results of complex interactions between invading pathogens and infected hosts. Upon presentation of the pathogen, the host innate immune response is initially elicited and subsequently regulated through interactions with intracellular adaptors, membrane-bound receptors, and intrinsic crosstalk among signaling pathways [1]. Many cytokines are produced in response to pathogen invasions, among them interferons (IFNs) are the most effective molecules in establishing multifaceted immune responses to limit the pathogen infections [2,3]. Three types of IFN (I, II, and III) are recognized, based on their structural features, receptor uses, and biological activities [4]. Most IFNs interact with specific receptors to trigger the Interferon/Janus kinase/Signal transducers and activators of transcription (IFN/JAK/STAT) signaling, which leads to the activation of IFN-stimulated genes (ISGs) [5].

Inductions of IFNs are mediated by the Toll-like receptor (TLR) family, and activations of TLRs allow associated adaptors to trigger downstream events [6,7]. Five Toll/interleukin-1 receptor (TIR)-specific adapters were identified. Myeloid differentiation primary response gene 88 (MyD88) is engaged by all TLRs except TLR3 [8]. TIR-domain-containing adaptor protein (TIRAP) is critical for TLR2 and TLR4 signaling [9,10]. TIR-domain-containing adapter-inducing IFN-α (TRIF) responds to TLR3 and TLR4 [11]. TRIF-related adaptor molecule (TRAM) is required for TLR4 signaling [12]. Sterile alpha and armadillo-motif containing protein (SARM) are engaged by TLR3 and TLR4 [13]. TIR-specific adapters subsequently activate signaling *via* either the MyD88-dependent pathway that stimulates the nuclear factor kappa-B (NF-κB) and induces the tumor necrosis factor-α (TNFα), interleukin-1 (IL-1), and IFNs, or MyD88-independent pathway that activates IFN regulatory factors (IRFs) and IFNs [14,15]. MyD88 recruits IL-1 receptor-associated kinase 1 (IRAK1), IRAK4, and TNF receptor-associated factor 6 (TRAF6) to form a complex [16]. IRAK4 subsequently induces IRAK1 and TRAF6 phosphorylation, which are dissociated from the complex to form a new one with transforming growth factor-activated kinase 1 (TAK1) and TAK1-binding protein 1/2 (Table 1/2) [17,18]. Activated TAK1 stimulates MAPK and IKKα/α, which phosphorylates IκBα and facilitates IκBα ubiquitination and degradation, leading to stimulation of NF-kB and induction of IFNs [19,20]. TLRs also trigger TRIF to activate the TANK-binding kinase 1 (TBK1) and TRAF6, leading to the activation of IRFs and NF-kB, which subsequently induces the expression of ISGs [21,22].

**Table 1.** Primers used in this study

Name of the primer	Sequence of the primer
Sox4-rqF	5ʹ-GTGAGCGAGATGATCTCGGG-3’
Sox4-rqR	5ʹ-CAGGTTGGAGATGCTGGACTC-3’
IFN-α-rqF	5ʹ-TTTCTCCTGCCTGAAGGACAG-3’
IFN-α-rqR	5ʹ-GCTCATGATTTCTGCTCTGACA-3’
IFN-β-rqF	5ʹ-TGGGAGGCTTGAATACTGCCTCAA-3’
IFN-β-rqR	5ʹ-TCCTTGGCCTTCAGGTAATGCAGA-3’
IFN-λ1 F-rqF	5ʹ-CTTCCAAGCCCACCACAACT-3’
IFN-λ1 R-rqR	5ʹ-GGCCTCCAGGACCTTCAGC-3’
TLR1-rqF	5ʹ-CAGTGTCTGGTACACGCATGGT-3’
TLR1-rqR	5ʹ-TTTCAAAAACCGTGTCTGTTAGAGA-3’
TLR2-rqF	5ʹ-GGCCAGCAAATTACCTGTGTG-3’
TLR2-rqR	5ʹ-AGGCGGACATCCTGAACCT-3’
TLR3-rqF	5ʹ-CCTGGTTTGTTAATTGGATTAACGA-3’
TLR3-rqR	5ʹ-TGAGGTGGAGTGTTGCAAAGG-3’
TLR4-rqF	5ʹ-CAGAGTTTCCTGCAATGGATCA-3’
TLR4-rqR	5ʹ-GCTTATCTGAAGGTGTTGCACAT-3’
TLR5-rqF	5ʹ-TGCCTTGAAGCCTTCAGTTATG-3’
TLR5-rqR	5ʹ-CCAACCACCACCATGATGAG-3’
TLR6-rqF	5ʹ-GAAGAAGAACAACCCTTTAGGATAGC-3’
TLR6-rqR	5ʹ-AGGCCAAACAAAATGGAAGCTT-3’
TLR7-rqF	5ʹ-TTTACCTGGATGGAAACCAGCTA-3’
TLR7-rqR	5ʹ-TCAAGGCCTGAGAAGCTGTAAGCTA-3’
TLR8-rqF	5ʹ-TTATGTGTTCCAGGAACTCAGAGAA-3’
TLR8-rqR	5ʹ-TAATACCCAAGTTGATAGTCGATAAGTTTG-3’
TLR9-rqF	5ʹ-GGACCTCTGGTACTGCTTCCA-3’
TLR9-rqR	5ʹ-AAGCTCGTTGTACACCCAGTCT-3’
TLR10-rqF	5ʹ-TGTTATGACAGCAGAGGGTGATG-3’
TLR10-rqR	5ʹ-GAGTTGAAAAAGGAGGTTATAGGATAAATC-3’
p65-rqF	5ʹ-CCTTCCAAGAAGAGCAGCGT-3’
p65-rqR	5ʹ-GATCTTGAGCTCGGCAGTGT-3’
p50-rqF	5ʹ-CCAACAGATGGCCCATACCT-3’
p50-rqR	5ʹ-AACCTTTGCTGGTCCCACAT-3’
IRF3-rqF	5ʹ-ACCAGCCGTGGACCAAGAG-3’
IRF3-rqR	5ʹ-TACCAAGGCCCTGAGGCAC-3’
IRF7-rqF	5ʹ-TGGTCCTGGTGAAGCTGGAA-3’
IRF7-rqR	5ʹ-GATGTCGTCATAGAGGCTGTTGG-3’
IAV-NP-vRNA-RT	5ʹ-CTCACCGAGTGACATCAACATCATG-3’
IAV-NP-cRNA-RT	5ʹ-AGTAGAAACAAGGGTATTTTTCTTTAATTGTCAT-3’
IAV-NP-rqF	5ʹ-ATCAGACCGAACGAGAATCCAGC-3’
IAV-NP-rqR	5ʹ-GGAGGCCCTCTGTTGATTAGTGT-3’
EV71-VP1-rqF	5ʹ-AATTGAGTTCCATAGGTG-3’
EV71-VP1-rqR	5ʹ-CTGTGCGAATTAAGGACAG-3’
GAPDH-rqF	5ʹ-GGAAGGTGAAGGTCGGAGTCAACGG-3’
GAPDH-rqR	5ʹ-CTCGCTCCTGGAAGATGGTGATGGG-3’

The genes detected by real-time PCR are listed. The sequences of each primer are presented. Sox4-rqF, real-time PCR forward primer for *Sox4* gene mRNA; Sox4-rqR, real-time PCR reverse primer for *Sox4* gene mRNA; IFN-α-rqF, real-time PCR forward primer for *IFN-α* gene mRNA; IFN-α-rqR, real-time PCR reverse primer for *IFN-α* gene mRNA; IFN-β-rqF, real-time PCR forward primer for *IFN-α* gene mRNA; IFN-β-rqR, real-time PCR reverse primer for *IFN-α* gene mRNA; IFN-λ1-rqF, real-time PCR reverse primer for *IFN-λ1* gene mRNA; IFN-λ1-rqR, real-time PCR reverse primer for *IFN-λ1* gene mRNA; TLR1-rqF, real-time PCR forward primer for *TLR1* gene mRNA; TLR1-rqR, real-time PCR reverse primer for *TLR1* gene mRNA; TLR2-rqF, real-time PCR forward primer for *TLR2* gene mRNA; TLR2-rqR, real-time PCR reverse primer for *TLR2* gene mRNA; TLR3-rqF, real-time PCR forward primer for *TLR3* gene mRNA; TLR3-rqR, real-time PCR reverse primer for *TLR3* gene mRNA; TLR4-rqF, real-time PCR forward primer for *TLR4* gene mRNA; TLR4-rqR, real-time PCR reverse primer for *TLR4* gene mRNA; TLR5-rqF, real-time PCR forward primer for *TLR5* gene mRNA; TLR5-rqR, real-time PCR reverse primer for *TLR5* gene mRNA; TLR6-rqF, real-time PCR forward primer for *TLR6* gene mRNA; TLR6-rqR, real-time PCR reverse primer for *TLR6* gene mRNA; TLR7-rqF, real-time PCR forward primer for *TLR7* gene mRNA; TLR7-rqR, real-time PCR reverse primer for *TLR7* gene mRNA; TLR8-rqF, real-time PCR forward primer for *TLR8* gene mRNA; TLR8-rqR, real-time PCR reverse primer for *TLR8* gene mRNA; TLR9-rqF, real-time PCR forward primer for *TLR9* gene mRNA; TLR9-rqR, real-time PCR reverse primer for *TLR9* gene mRNA; TLR10-rqF, real-time PCR forward primer for *TLR10* gene mRNA; TLR10-rqR, real-time PCR reverse primer for *TLR10* gene mRNA; p65-rqF, real-time PCR forward primer for *p65* gene mRNA; p65-rqR, real-time PCR reverse primer for *p65* gene mRNA; p50-rqF, real-time PCR forward primer for *p50* gene mRNA; p50-rqR, real-time PCR reverse primer for *p50* gene mRNA; IRF3-rqF, real-time PCR forward primer for *IRF3* gene mRNA; IRF3-rqR, real-time PCR reverse primer for *IRF3* gene mRNA; IRF7-rqF, real-time PCR forward primer for *IRF7* gene mRNA; IRF7-rqR, real-time PCR reverse primer for *IRF7* gene mRNA; IAV-NP-vRNA-reverse transcription, reverse transcription primer for IAV *NP* gene vRNA; IAV-NP-cRNA-reverse transcription, reverse transcription primer for IAV *NP* gene cRNA; IAV-NP-rqF, real-time PCR forward primer for IAV *NP* gene mRNA; IAV-NP-rqR, real-time PCR reverse primer for IAV *NP* gene mRNA; EV71-VP1-rqF, real-time PCR forward primer for EV71 *VP1* gene mRNA; EV71-VP1-rqR, real-time PCR reverse primer for EV71 *VP1* gene mRNA; GAPDH-rqF, real-time PCR forward primer for *GAPDH* gene mRNA; GAPDH-rqR real-time PCR reverse primer for GAPDH gene mRNA.

Given the critical role of TLR in innate immunity, it is logical that TLR signaling is targeted by the pathogens in order to evade host immunity. Many cellular factors have been identified to repress TLR signaling through different mechanisms, including extracellular, transmembrane, and intracellular regulations and protein degradation [23–25]. TLR signaling is also negatively regulated by microRNAs (miRNAs), including miRNA-146b [26] and miRNA-21 [27]. However, they regulate TLR pathways only by targeting a single molecule or a shared molecule at one stage in the signaling network. Here, we demonstrated that the sex-determining region Y-box 4 (Sox4) acts as a master regulator to hijack TLR networks at multiple stages and facilitate pathogen infection by a unique mechanism.

Sox4 belongs to the SRY-related HMG box (Sox) family that comprises 20 members in human and mouse [28]. It contains a conserved high-mobility group (HMG) that binds preferentially to the targeted genes, and a transactivation domain (TAD) that activates gene transcription [29,30]. Sox4-deficient mice are embryonic lethality due to cardiac defects, whereas heterozygous and mutant mice suffer from multiple developmental defects [31,32]. Sox4 is important for the development of multiple tissues and organs, and is associated with the development of many cancers [33–36]. Recently, we at the first time reported that Sox4 production and hepatitis B virus (HBV) replication are tightly controlled by a novel positive feedback mechanism [37]. However, the roles of Sox4 in the regulation of host immunity and TLR signaling are still unknown. In this study, we initially showed that Sox4 expression is induced during the infections of enterovirus 71 (EV71), influenza A virus (IAV), hepatitis C virus (HCV), and vesicular stomatitis virus (VSV). More interestingly, Sox4 subsequently facilitates viral replications by repressing the entire TLR signaling networks at multiple stages to shut down host immunity. Therefore, we revealed a distinct mechanism underlying global control of host immunity and pathogen infection.

## Materials and Methods

### Human clinical specimens

Clinical throat swab specimens from 27 influenza A virus (IAV)-infected patients and 20 healthy individuals were collected from Wuhan Children’s Hospital (Wuhan, China). The patients were not suffering from any concomitant illnesses and did not express any serological markers suggestive of autoimmune disease. All specimens were treated with TRIzol reagent according to the protocol provided by the manufacturer (Invitrogen). Total RNAs of the specimens were extracted and reverse transcribed to cDNA using random primers and IAV *NP* gene reverse primer. Levels of the sex-determining region Y-box 4 (*Sox4*) mRNA and IAV *NP* mRNA were detected by real-time PCR.

Blood samples of healthy donors were collected from Wuhan General Hospital of Guangzhou Military (Wuhan, China). To isolate peripheral blood mononuclear cells (PBMCs), blood cells were separated from blood samples and diluted in RPMI-1640 purchased from Gibco (Grand Island, NY, USA). Diluted blood cells (5 ml) were added gently to a 15 ml centrifuge tube with 5 ml lymphocyte separation medium (#50,494) purchased from MP Biomedicals (California, USA), and centrifuged at 2,000 × *g* for 10 min at room temperature (RT). The middle layer was transferred to another new centrifuge tube and diluted with RPMI-1640. The remaining red blood cells were removed using red blood cell lyses buffer purchased from Sigma-Aldrich (St. Louis, MO, USA). The pure PBMCs were centrifuged at 1,500 × *g* for 10 min at RT and cultured in RPMI-1640.

The study was conducted according to the principles of the Declaration of Helsinki and approved by the Institutional Review Board of the College of Life Sciences, Wuhan University, in accordance with its guidelines for the protection of human subjects. All participants provided written informed consent to participate in the study.

## Cells and cultures

Human hepatoma cell line (HepG2), human hepatoma cell line (Huh7), normal liver cell line (L02), human lung epithelial cell line (A549), human rhabdomyosacroma cell line (RD), and human embryonic kidney cell line (HEK 293 T) were purchased from American Type Culture Collection (ATCC) (Manassas, VA, USA). Human hepatoma cell line (Huh7.5.1) was kindly provided by Dr. Francis V Chisari of Scripps Research Institute, USA. Cells were cultured in DMEM (Dulbecco’s modified Eagle medium) purchased from Gibco (Grand Island, NY, USA) supplemented with 10% heat-inactivated FBS (fetal bovine serum) (Gibco) at 37°C in a humidified atmosphere of 5% CO_2._

## Viruses and infections

Human enterovirus 71 (EV71) strain (Xiangyang-Hubei-09) was isolated previously by our group (GenBank accession no. JN230523.1) and the virus stock was propagated in RD cells [38]. Sendai virus (SeV) strain was a gift from Dr. Hongbing Shu of Wuhan University. The Indiana serotype of Vesicular stomatitis virus (VSV) strain was provided by China Center Type Culture Collection (CCTCC) (Wuhan, China). Recombinant GFP-VSV strain expressing green fluorescent protein was a gift from Dr. Mingzhou Chen of Wuhan University. L02 Cells were infected with GFP-VSV at a multiplicity of infection (MOI) of 2 (MOI = 2) and the unbound virus was washed away 2 h later, as described previously [39]. Influenza A virus (IAV) strain A/HongKong/498/97 (H3N2) was provided by CCTCC (Wuhan, China). The virus stock was propagated in A549 cells cultured in F12K medium (Invitrogen), as described previously [41]. Hepatitis C virus (HCV) genotype 2a strain JFH-1 was kindly provided by Dr. Takaji Wakita of the National Institute of Infectious Diseases. Huh7 cells and Huh7.5.1 cells were infected with JFH-1 at MOI = 1, as described previously [27]. The growth, virus titration, and inoculation of EV71, SeV, VSV, IAV, and HCV were performed as described previously [37,40].

## Plasmids and constructions

The CD (coding sequence) of *Sox4* (NM_00317.2) and the two deletion mutants *Sox4ΔHMG* and *FLAG-Sox4ΔTAD* were subcloned into pCMV-tag2A to generate pFLAG-Sox4, pFLAG-Sox4ΔHMG, and pFLAG-Sox4ΔTAD, respectively. The CDS of ubiquitin was subcloned into pcDNA3.1-Myc to generate pMyc-Ub, as described previously [37].

pFLAG-TLR3, pFLAG-TLR4, pFLAG-TLR7, and pFLAG-TLR9 were purchased from Beijing Zhongyuan, Ltd. (Beijing, China). pFLAG-p65, pFLAG-p50, pFLAG-IRF3, pFLAG-IRF7, pGFP-p65, pGFP-p50, pGFP-IRF3, pGFP-IRF7, pIFN-β-Luc, and pISRE-Luc were kindly provided by Dr. Ying Zhu of Wuhan University. The full-length promoters of *TLR1, TLR3, TLR4, TLR5*, and *TLR7* were subcloned into pGL3-Basic to generate pTLR1-Luc, pTLR3-Luc, pTLR4-Luc, pTLR5-Luc, and pTLR7-Luc, respectively. All plasmids were confirmed by sequencing analysis and the resulting proteins were verified by Western blotting.

## Reagents

Antibodies against Mx1 (sc-398,564), PKR (sc-707), OAS1 (sc-98,424), TLR5 (sc-10,742), and TLR10 (sc-30,198) were purchased from Santa Cruz Biotechnology (Santa Cruz, CA, USA). Antibodies against TLR1 (#2209), TLR2 (#2229), TLR3 (#2253), TLR4 (#2246), TLR6 (#12,717), TLR7 (#2633), TLR8 (#11,886), TLR9 (#2254), ubiquitin (#3933), MYD88 (#4283S), IRAK4 (#4363), and IRAK1 (#4504S), and NF-kB Pathway Sampler Kit, were purchased from Cell Signaling Technology (Beverly, MA, USA). Antibodies against FLAG (F3165) and GFP (G1546) were purchased from Sigma-Aldrich (St. Louis, MO, USA).

siRNAs specific to *Sox4* (siR-Sox4) were designed and synthesized by RiboBio (Guangzhou, China) and tested in a previous report [37]. TNF-α (T0157), CHX (N11534), BAY11-7082 (B5556), and the proteasome inhibitor MG-132 (M7449) were purchased from Sigma-Aldrich (St. Louis, MO, USA). Complete Protease Inhibitor Cocktail Tablets and PhosSTOP Phosphatase Inhibitor Cocktail Tablets were purchased from Roche (Basel, Switzerland).

## RNA extraction and real-time RT-PCR

Total RNA was extracted from cells or transfected cells using TRIzol reagent according to the protocol provided by the manufacturer (Invitrogen). DNA was removed from the sample using on-column DNase I treatment at 37°C for 30 min. RNA was washed with 75% ethanol and redissolved in DEPC ddH_2_O. The concentration and quality of RNA were measured using NanoDrop 2000 (Thermo Scientific, MA, USA). RNA (1 μg) was used as a template to synthesize cDNA using random primers (2.5 μM, 1 μl) and moloney murine leukemia virus (MMLV) reverse transcriptase (1 μl) (Promega, Madison, WI, USA) at 42°C for 60 min, which was then denatured for 10 min at 75°C; the total reaction volume was 20 μl.

Real-time PCR (RT-PCR) was performed using SYBR Green PCR master mix in a Light Cycler 480 (Roche Diagnostics Ltd., Risch-Rotkreuz, Switzerland). After an initial incubation at 95°C for 5 min, the reaction mixtures were subjected to 40 cycles of amplification under the following conditions: 94°C for 15 s, 56°C for 15 s, and 72°C for 20 s. The fluorescence was measured at this step to assess the quality of the primers, which was followed by a final melting curve step from 50°C to 95°C. Each sample was run in triplicate, and the threshold cycles (Cts) were averaged and normalized to endogenous glyceraldehyde 3-phosphate dehydrogenase (GAPDH). The relative amount of amplified product was calculated using the comparative Ct method. The primers used in this study are listed in Table 2.

**Table 2.** Primers of real-time PCR used in this study for genes detections

Gene	Location of the Sox4 binding site	Sequence of the Sox4 binding site on corresponding TLR promoters
TLR1	−376 p to −370 bp	5ʹ-GGCAAGAGGAAAAACAAAGCAGCCGAAACA-3’
TLR2	none	none
TLR3	+197 bp to +205 bp	5ʹ-TCTTTGGTCTTTCTTTGATCTGGTGCTTGAAAT-3’
TLR4	−997 bp to −990 bp -124 bp to −118 bp	5ʹ-AGGAAGGAGGCTTTGATCTATACTACACAG-3ʹ 5ʹ-CCACAGCTGAACAAAATGGAAAATCAC-3’
TLR5	−1792 bp to −1786 bp -865 bp to −859 bp	5ʹ-AGAATATAAACTTTGTTTGTAGTTCATAGG-3ʹ 5ʹ-TAAGTGCCAGGCTTTGTTTTACACCTATCT-3’
TLR6	−1363 bp to −1357 bp -677 bp to −670 bp	5ʹ-TACTGTCTGTTTCTTTGTTTGCTTAACTGT-3ʹ 5ʹ-TCTGGTAATCAGCCTTTGTTGATGTCATTCT-3’
TLR7	−1937 bp to −1930 bp -78 bp to −71 bp	5ʹ-GTAATGCACCCTTTGTTATATGAAAGGAG-3ʹ 5ʹ-CCGACCTGATCTTTGTAGTTGGAAACT-3’
TLR8	−406 bp to −400 bp	5ʹ-CAGAAACTTGTGGAACAAAGATGAAGCA-3’
TLR9	−761 bp to −754 bp	5ʹ-AAAAAATGTTAGGACAAAGAGAAACATAGA-3’
TLR10	−213 bp to −207 bp +83 bp to +89 bp	5ʹ-CCAGCCTGGGTGACAAAGTGAGACCCTACC-3ʹ 5ʹ-GTAAGAACCTTAGCTTTGTTTGTTGTAACT-3’

Sequence analyses revealed that the promoters of all *TLRs* genes (excluding *TLR2* gene) contain one or two potential Sox4 binding sites. The locations of Sox4 binding sites are indicated. The sequences of Sox4 binding sites on corresponding *TLR* promoters are presented. The core sequences of the Sox4 binding sites are underlined.

## Western blotting

Cells were harvested at 48 h post-transfection or at the indicated treatment times, washed once with ice-cold phosphate-buffered saline (PBS), and re-suspended in lyses buffer (20 mM HEPES, 150 mM NaCl, 1 mM EDTA, 1 mM EGTA, and 1% Triton-100, pH7.5) supplemented with 1 × protease inhibitor cocktail (Roche, Basel, Switzerland). Lysates were sonicated on ice and then centrifuged at 10,000 × *g* for 5 min at 4°C to remove cell debris. Then the concentration of protein in each sample was determined using a Bradford assay kit (Bio-Rad, Hercules, CA, USA). Cell lysates (100 μg) were electrophoresed using 10% sodium dodecyl phosphate–polyacrylamide gel electrophoresis (SDS-PAGE) and transferred to nitrocellulose membranes (Amersham, Milwaukee, WI, USA). Nonspecific sites were blocked using 5% nonfat dried milk in PBS containing 0.05% Tween 20 (PBST) for at least 1 h at RT, and then the membranes were incubated with specific primary antibodies for at least 3 h at RT or overnight at 4°C. The bound primary antibodies were detected by incubation with the appropriate secondary antibodies for 45 min at RT. The blots were analyzed using a luminescent image analyzer (LAS-4000; Fujifilm, Tokyo, Japan).

## Dual-luciferase reporter assay

For dual-luciferase assays, the expression of Renilla luciferase was used as a reference under the control of an independent cytomegalovirus (CMV) promoter, and that of firefly luciferase was under the control of the inserted target gene’s promoter. The ratio of firefly luciferase activity to Renilla luciferase activity reflects the final relative luciferase activity for each sample. Cells were transfected with the indicated plasmids and luciferase reporter plasmid for 48 h, after which the cells were washed twice with ice-cold PBS. Luciferase lyses buffer (100 µl) (Promega, Madison, Wisconsin, USA) was added to each well of a 24-well plate. Cells were lysed for 10 min at RT, after which 50 µl of each sample was transferred to a new centrifuge tube and mixed with 15 µl of the corresponding luciferase assay substrate (Promega, Madison, Wisconsin, USA). Luciferase activity was typically measured for 10 s using a luminometer (TD-20/20; Turner Designs, Sunnyvale, CA, USA). All assays were performed in triplicate, and the data are expressed as means ± SD (standard deviation).

## Co-immunoprecipitation (Co-IP)

Human embryonic kidney (HEK293T) cells were seeded in dishes (10 cm diameter) and co-transfected with the indicated plasmids for 2 days. Cells were lysed using radioimmunoprecipitation assay (RIPA) buffer (20 mM Tris-HCl, pH7.4, 150 mM NaCl, 1 mM EDTA, and 1% Triton-X-100 supplemented with protease inhibitors. Lysates were sonicated on ice and whole cell extracts (WCEs) were centrifuged at 10,000 × *g* for 5 min at 4°C to remove cell debris. One-fourth of the supernatant was used as input. Then the remaining supernatants were collected, pre-cleared using protein G Sepharose beads (GE Healthcare, Milwaukee, WI, USA), and incubated with the indicated antibodies overnight at 4°C. The supernatants were then mixed with protein G sepharose beads (GE Healthcare, Milwaukee, WI, USA) for 2 h at 4°C. The immunoprecipitates were centrifuged at 2,000 × *g* for 2 min at 4°C, washed five times with RIPA lyses buffer, eluted with 1% SDS buffer, boiled in loading buffer for 5 min, and then analyzed using SDS-PAGE and Western blotting.

## Chromatin immunoprecipitation assay (ChIP)

Cells were seeded in dishes (10 cm diameter) and transfected with the indicated plasmids. Chromatin immunoprecipitation (ChIP) assays were performed according to the X-ChIP protocol (Abcam). Formaldehyde was added to the culture medium to a final concentration of 1% for 5 min at RT and then a final concentration of 125 nM glycine was added to stop the cross-linking reaction for 5 min at RT. The cells were washed twice with ice-cold PBS, scraped, centrifuged, and lysed in ChIP lyses buffer (50 mM HEPES-KOH, pH7.5, 140 mM NaCl, 1 mM EDTA, 1% Triton X-100, 0.1% sodium deoxycholate, 0.1% SDS, and protease inhibitors). Lysates were sonicated on ice and the debris was removed by centrifugation. One-fourth of the supernatant was used as DNA input. The remaining supernatant was diluted 10-fold with dilution buffer, pre-cleared using protein G Sepharose beads (GE Healthcare, Milwaukee, WI, USA), and incubated with the indicated antibodies overnight at 4°C. The supernatant was then mixed with protein G Sepharose beads (GE Healthcare, Milwaukee, WI, USA) for 2 h at 4°C. Immunoprecipitated complexes were centrifuged at 2,000 × *g* for 2 min at 4°C for collection, washed with dialysis buffer, and eluted with elution buffer (1% SDS and 100 mM NaHCO_3_). Then the resulting supernatant was incubated at 67°C for 5 h to reverse formaldehyde cross-linking with a final concentration of 0.1 M NaCl, and proteins were removed by adding proteinase K for 1 h at 45°C. DNA was precipitated with ethanol and extracted three times with phenol/chloroform. Pellets were re-suspended in TE buffer and subjected to PCR amplification using the corresponding primers.

## Cytoplasm and nucleus isolation

Cells were seeded in six-well plates and transfected with the indicated plasmids. At 48 h post-transfection, cells were washed twice with ice-cold PBS, collected, and lysed in two volumes of buffer A (10 mM HEPES, pH8.0, 0.5% Nonidet P-40, 1.5 mM MgCl_2_, 10 mM KCl, 0.5 mM dithiothreitol, and 200 mM sucrose) for 15 min at 4°C with tube flipping. A final concentration of 0.5% NP-40 was then added to the lysates with tube flipping for 5 s and the lysates were centrifuged at 16,000 × *g* for 5 min at 4°C. The supernatant, as cytoplasm extract, was transferred to a fresh tube. The sediment was rinsed with buffer A, resuspended in one volume of buffer B (20 mM HEPES, pH7.9, 1.5 mM MgCl_2_, 420 mM NaCl, 0.2 mM EDTA, and 1.0 mM DTT) with tube flipping for 15 s, and incubated on a shaking platform for 30 min at 4°C. The nuclei were centrifuged at 16,000 × *g* for 5 min at 4°C and the supernatants were collected. Cocktail protease inhibitor was added to each buffer. The cytoplasm and nuclear extracts were stored at −80°C until use.

## Protein degradation and ubiquitination assays

HEK293T cells and Human liver carcinoma (HepG2) cells were co-transfected with the indicated plasmids for 36 h and treated with the protein synthesis inhibitor, cycloheximide (CHX), at a final concentration of 50 μg/ml for the indicated times before harvest. Cells were lysed in Western blot lyses buffer with proteinase inhibitor cocktail. Lysates were sonicated on ice and the debris was removed by centrifugation. Then whole cell extracts were prepared as described above and were used for Western blotting.

For ubiquitination assays, HEK293T cells were seeded in dishes (10 cm diameter), co-transfected with the indicated plasmids for 36 h, and treated with the proteasome inhibitor MG-132 at a final concentration of 20 μM for 9 h. Cells were lysed in RIPA buffer with proteinase inhibitor cocktail and sonicated gently three times on the ice. Cell lysates were centrifuged to remove the debris and the supernatants were divided into two aliquots: one aliquot (5%) was used as a whole cell extract for Western blotting, while the other (95%) was incubated with the indicated antibodies overnight at 4°C and then incubated with protein G Sepharose beads for 2 h at RT. The precipitates were washed five times with RIPA buffer and then the bound proteins were eluted with 1% SDS buffer, boiled in loading buffer for 5 min, and analyzed using SDS-PAGE and Western blotting.

## Statistical analyses

All experiments were reproducible and repeated at least three times with similar results. Parallel samples were analyzed for normal distribution using Kolmogorov-Smirnov tests. Abnormal values were eliminated using a follow-up Grubbs test. Levene’s test for equality of variances was performed, which provided information for Student’s *t*-tests to distinguish the equality of means. Means were illustrated using histograms with error bars representing the SD; a *P* value of <0.05 was considered statistically significant.

## Results

### Viral infections activate Sox4 expression to facilitate viral replications

**Figure 1.**
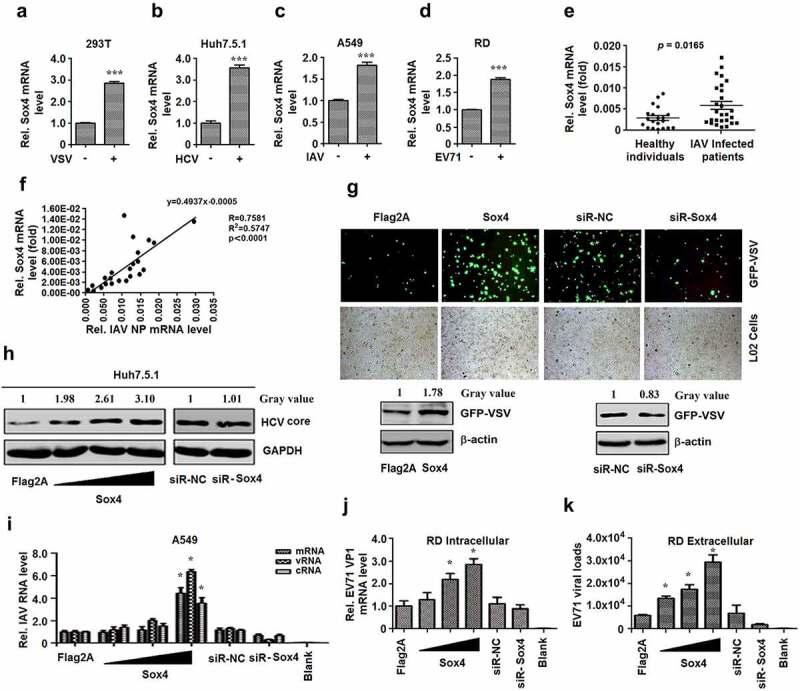
**Viruses activate Sox4 expression, which subsequently facilitates viral replication**. (**A** to **D**) 293 T cells were infected with VSV (a), Huh7.5.1 cells were infected with HCV (b), A549 cells were infected with IAV (c), RD cells were infected with EV71 (d). *Sox4* mRNAs expressed in infected cells were detected by RT-PCR. (e and f) *Sox4* mRNAs expressed in IAV-infected patients and healthy individuals were detected by RT-PCR (e). *Sox4* and IAV *NP* mRNAs expressed in IAV-infected patients were detected by RT-PCR, and the correlations of *Sox4* mRNAs and IAV *NP* mRNAs were analyzed (f). (g) Fluorescence micrographs of L02 cells infected with GFP-VSV and transfected with pFlag-Sox4 or siR-Sox4 (upper panel). Western Blot analysis the replication of GFP-VSV corresponding to the cells for acquiring micrographs by anti-GFP (lower panel). (h) Huh7.5.1 cells were infected with HCV and transfected with pFlag-Sox4 in a dose-dependent manner (left) or siR-Sox4 (right). HCV core proteins were detected by Western blots. (i) A549 cells were infected with IAV and transfected with pFlag-Sox4 or siR-Sox4. IAV *NP* mRNA, vRNA, and cRNA were analyzed by RT-PCR. (j and k) RD cells were infected with EV71 and transfected with pFlag-Sox4 or siR-Sox4. HCV *VP1* mRNAs in the cell extractions (j) or culture supernatants (k) were determined by RT-PCR. Results are shown as means ± SD (n = 3). *P < 0.05, ***P < 0.001

Sox4 is a multi-functional regulator involved in cell development, differentiation, and tumorigenesis. Here, we determined the roles of Sox4 in the regulation of host immunity and pathogen infection. The correlation between viral infection and Sox4 function was initially evaluated. We showed that *Sox4* mRNA was induced in human embryonic kidney cells (HEK 293 T) infected with vesicular stomatitis virus (VSV), human hepatoma cells (Huh7.5.1) infected with hepatitis C virus (HCV), human lung adenocarcinoma cells (A549) infected with influenza A virus (IAV), and human rhabdomyosarcoma cells (RD) infected with enterovirus 71 (EV71) (Figure 1a–d). Moreover, the average *Sox4* mRNAs were significantly higher in IAV-infected patients as compared to healthy individuals (Figure 1e), and the levels of *Sox4* mRNAs and IAV *NP* mRNAs in patients were positively correlated (R = 0.7581) (figure 1f). Therefore, *Sox4* is activated during the infections of VSV, HCV, IAV, and EV71.

As Sox4 is a transcriptional factor, we want to evaluate if the activated Sox4 by viral infection could in turn have functions in the regulation of viral replication. VSV, HCV, and IAV replication in vitro were separately measured with or without Sox4. Firstly, VSV replication was enhanced by Sox4 and reduced by siR-Sox4 in L02 cells infected with recombinant green fluorescent protein-VSV (GFP-VSV). Similarly, viral protein expression was upregulated by Sox4 and downregulated by siR-Sox4 in GFP-VSV-infected cells (Figure 1g). In addition, HCV core protein was elevated by Sox4 and reduced by siR-Sox4 in HCV-infected cells (Figure 1h). The three types of IAV RNA, messenger RNA (mRNA), viral RNA (vRNA), and complementary RNA (cRNA), were enhanced by Sox4 and reduced by siR-Sox4 in IAV-infected cells (Figure 1i). Finally, EV71 *VP1* mRNA in intracellular extracts of infected cells and viral load in extracellular extracts of infected cells were stimulated by Sox4 and inhibited by siR-Sox4 (Figure 1j and K). These results suggested that Sox4 facilitates VSV, HCV, IAV, and EV71 replication. Taken together, we demonstrated that virus infection activates Sox4, which in turn facilitates viral replications.

## Sox4 attenuates IFNs and ISGs production

**Figure 2.**
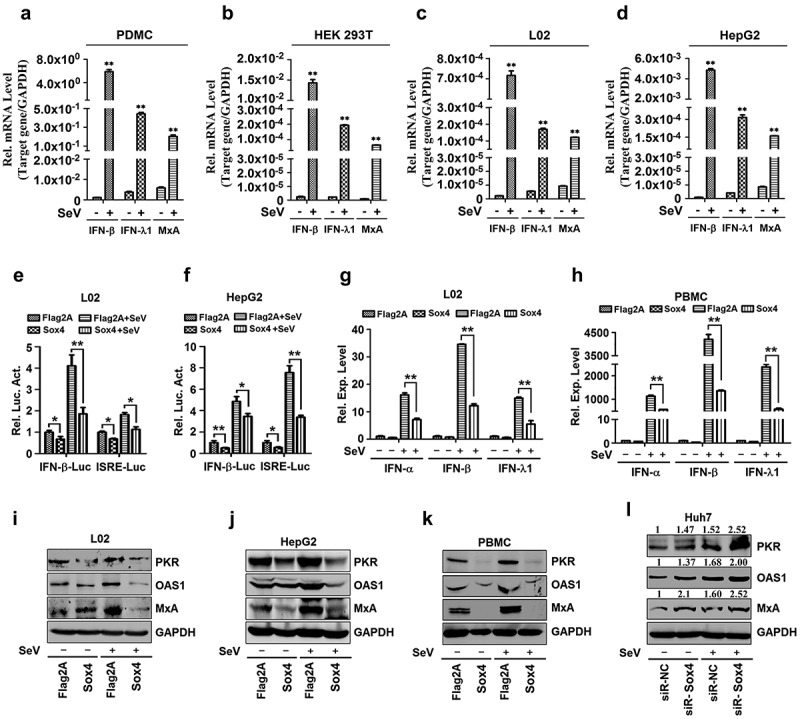
**Sox4 attenuates the expressions of IFNs and ISGs during viral infections**. (a) Human peripheral blood mononuclear cells (PBMCs) were infected with or without SeV for 12 h. Total mRNA extractions were prepared from the infected cells and *IFN-β, IFN-λ1*, and *Mx1* mRNAs were determined by real-time PCR. **P < 0.01. (b) Human embryonic kidney (HEK293T) cells were infected with or without SeV for 12 h. Total mRNA extractions were prepared from the infected cells and *IFN-β, IFN-λ1*, and *Mx1* mRNAs were determined by real-time PCR. **P < 0.01. (c) Human hepatocyte cells (L02) were infected with or without SeV for 12 h. Total mRNA extractions were prepared from the infected cells and *IFN-β, IFN-λ1*, and *Mx1* mRNAs were determined by real-time PCR. **P < 0.01. (d) Human liver carcinoma cells (HepG2) were infected with or without SeV for 12 h. Total mRNA extractions were prepared from the infected cells and *IFN-β, IFN-λ1*, and *Mx1* mRNAs were determined by real-time PCR. **P < 0.01. (e and f) L02 cells (e) or HepG2 cells (f) were co-transfected with pFlag-Sox4 or pFlag2A and pIFN-β-Luc or pISRE-Luc, and then infected with SeV. Luciferase activities in the cell extracts were measured by using a TD-20/20 luminometer. (g and h) L02 cells (g) or human peripheral blood mononuclear cells (PBMCs) (h) were transfected with pFlag-Sox4 or pFlag2A and infected with SeV. The levels of *IFN-α, IFN-α*, and *IFN-λ1* mRNAs expressed in the cells were determined by RT-PCR. (i–k) L02 cells (i), HepG2 cells (j), or PBMCs (k) were transfected with pFlag-Sox4 or pFlag2A and infected with SeV. (l) Human hepatoma cells (Huh7) were transfected with siR-Sox4 or sir-NC and infected with SeV. The levels of PKR, OAS1, Mx1, and GAPDH proteins expressed in the cells were detected by Western blot analyses using corresponding antibodies, as indicated. The results are presented as means ± SD (n = 3). *P < 0.05, **P < 0.01

Since Sox4 plays a role in the facilitation of so many viruses replications, it is more like a wide spectrum promotion on viral replication than a specific function. Thus, we speculated that Sox4 may regulate innate immunity. To confirm this speculation, firstly, we tested if the cell lines we used could be stimulated by stimulus (SeV is used here as a stimulus). The activation of *IFN-β* (a type I IFN), *IFN-λ1* (a type III IFN), and IFN-induced GTP-binding protein (*MxA*) (an ISG) is then evaluated during SeV infection. The results indicated that all the cell lines used had activated IFN-pathway and were suitable for this study (Figure 2a–d). Then, the function of Sox4 on IFN pathway activation was performed. The results showed that Sox4 obviously suppressed Sev stimulated activities of *IFN-β* promoter and IFN-stimulated response element (*ISRE*) in L02 and HepG2 cells (Figure 2e and f). In addition, SeV induced *IFN-α, IFN-β*, and *IFN-λ1* expression were also attenuated by Sox4 in L02 and PBMCs (Figure 2g and h). Moreover, three typical ISGs, double-strand RNA-activated protein kinase (*PKR*), 2ʹ,5ʹ-oligoadenylate synthetase 1 (*OAS1*), and *Mx1*, were repressed by Sox4 in L02, HepG2, and PBMCs during SeV stimulation (Figure 2i–k), but enhanced by siR-Sox4 in infected Huh7 cells (Figure 2l). Therefore, we confirmed that Sox4 represses IFN pathway activation.

## Sox4 represses NF-kB signaling by interacting with IKKα/α complex

**Figure 3.**
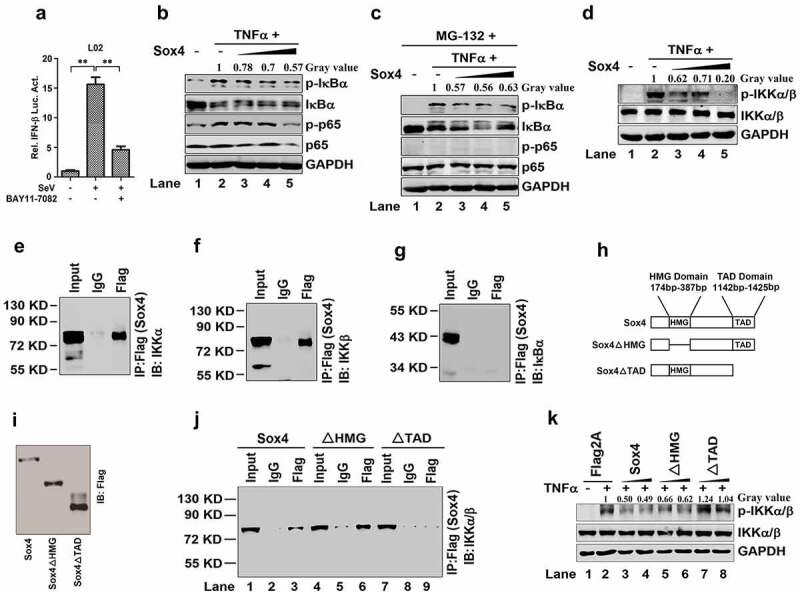
**Sox4 inhibits NF-kB activity through interacting with IKKα/α** (a) L02 cells were transfected with pIFN-β-Luc for 24 h, treated with BAY11-7082 (a specific inhibitor of NF-kB) for 9 h, and then infected with SeV for 12 h. Luciferase activities were measured using a TD-20/20 luminometer and normalized to the control. The results are presented as means ± SDs (*n* = 3). **P < 0.01. (b and c) L02 cells were transfected with pFlag-Sox4 or pFlag2A and treated with TNFα (b) or treated with MG-132 and then treated with or without TNFα (c). The p-IkBα, IkBα, p-p65, p65, and GAPDH proteins expressed in the cells were detected by Western blot analyses. (d) L02 cells were transfected with pFlag-Sox4 or pFlag2A and treated with TNFα. The p-IKKα/α, IKKα/α, and GAPDH proteins expressed in the cells were detected by Western blot analyses. (e–g) HEK293T cells were transfected with pFlag-Sox4. Co-IP assays for the transfected cells were performed using antibody to Flag, and the precipitates were analyzed using antibody to IKKα (e), using antibody to IKKβ (f), or using antibody to IκBα (g). (h) Diagrams of the wild-type Sox4 protein and its two mutants, Sox4∆HMG and Sox4∆TAD. In Sox4∆HMG, the HMG domain of Sox4 was deleted, whereas in Sox4∆TAD, the TAD domain of Sox4 was deleted. (i) HEK293T cells were transfected with pFlag-Sox4, pFlag-Sox4∆HMG, or pFlag-Sox4∆TAD for 48 h. The cells were collected and lysed in Western bolt lyses buffer. Sox4, Sox4∆HMG, and Sox4TAD proteins were analyzed by Western blot using antibodies specific to FLAG. (j) HEK293T cells were transfected with pFlag-Sox4, pFlag-Sox4ΔHMG, and pFlag-Sox4ΔTAD, respectively. Co-IP assays were conducted using antibody to Flag, and the precipitates were analyzed using antibody to IKKα/β. (k) L02 cells were transfected with pFlag2A, pFlag-Sox4, pFlag-Sox4ΔHMG, and pFlag-Sox4ΔTAD, respectively, and treated with TNFα. The p-IKKα/α, IKKα/α, and GAPDH proteins expressed in the cells were detected were detected by Western blot analyses

The mechanism underlying Sox4-mediated repression of IFNs and ISGs was then investigated. As NF-kB is a key regulator for the activation of IFNs, we firstly determined whether NF-kB is involved in Sox4-mediated regulation of IFNs. The activation of *the IFN-β* promoter stimulated by SeV could be repressed by a specific inhibitor of NF-kB (BAY11 7082) (Figure 3a), suggesting that NF-kB is a key regulator for the IFN pathway as reported. Since NF-kB needs to be activated by phosphorylation of the subunits and the phosphorylation of p65 (p-p65) needs phosphorylation related degradation of IkBα, we evaluated the role of Sox4 on the phosphorylation states of IkBα and NF-kB p65 induced by TNFα. Sox4 could reduce all the phosphorylation states of IkBα and NF-kB p65 (Figure 3b), indicating Sox4 represses NF-kB activation. p-p65 was entirely inhibited in the presence of proteasome inhibitor (MG-132) (Figure 3c), indicating there is no phosphorylation-related degradation of p-IkBα. However, p-IkBα stimulated by TNFα was still repressed by Sox4 in the presence of MG-132 (Figure 3c), suggesting that Sox4 represses NF-kB activity by inhibiting IkBα phosphorylation. Since phosphorylation of IkBα is dependent on IKKα/β [20], we then examined the role of Sox4 in the regulation of IKKα/β. Phosphorylation of IKKα/β was stimulated by TNFα and attenuated by Sox4 (Figure 3d), demonstrating that Sox4 represses NF-kB activity through inhibiting activation of IKKα/β.

Sox4 contains an HMG domain that binds to targeted genes and a TAD domain that activates targeted genes [42]. We speculated that Sox4 may inhibit IKKα/β by binding with the complex. Co-IP assays confirmed that Sox4 could bind with IKKα and IKKβ, but not with IκBα (Figure 3e–g). To map the domain of Sox4 required for interaction with IKKα/β, we constructed two mutants of Sox4 (Sox4ΔHMG and Sox4ΔTAD), in which the HMG or TAD domain was deleted (Figure 3h), and the expression of Sox4 and the two mutant proteins was confirmed (Figure 3i). Co-IP assays revealed that Sox4 and Sox4ΔHMG could interact with IKKα/β, but Sox4ΔTAD failed to act (Figure 3j). In addition, IKKα/β phosphorylation activated by TNFαwas repressed by Sox4 and Sox4ΔHMG, but not by Sox4ΔTAD (Figure 3k). These results demonstrated that Sox4 binds with the IKKα/β complex and inhibits IKKα/β phosphorylation through its TAD domain, which leads to the repression of NF-kB activation.

## Sox4 attenuates NF-kB and IRF3/7 by facilitating protein degradation

**Figure 4.**
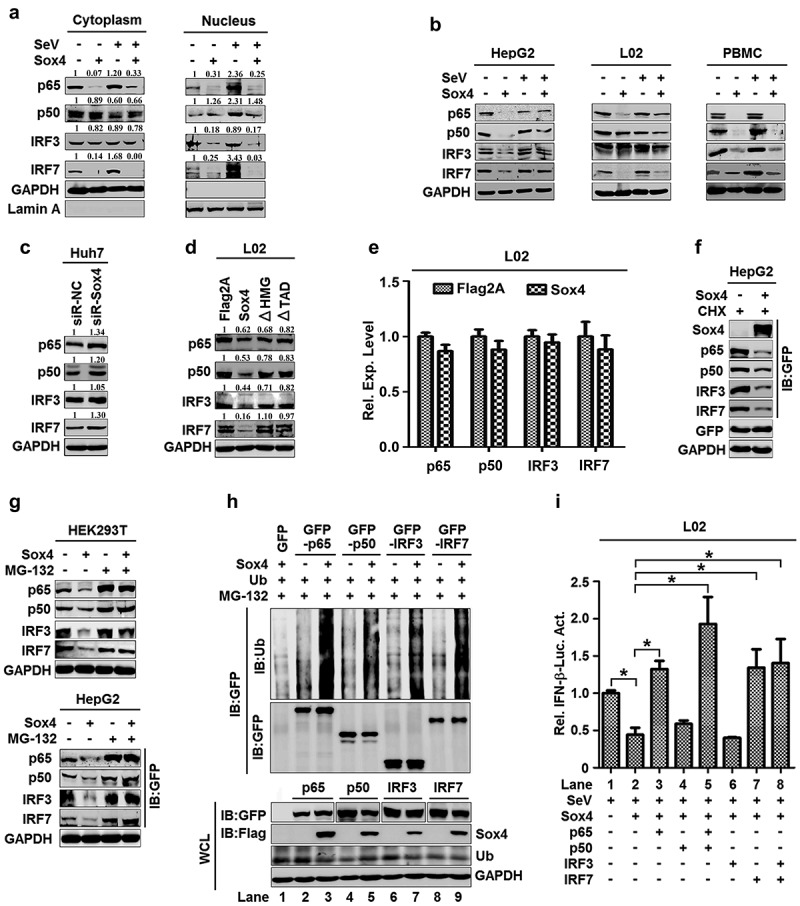
**Sox4 downregulates NF-kB and IRF3/7 by facilitating protein degradation**. (a) L02 cells were transfected with pFlag-Sox4 and infected with SeV. Proteins expressed in the cytoplasm (left) or nucleus (right) were detected by Western blot analyses. (b–f) HepG2 cells, L02 cells, and PBMCs were transfected with pFlag-Sox4 and infected with SeV (b). Huh7 cells were transfected with siR-Sox4 (c). L02 cells were transfected with pFlag-Sox4, pFlag-Sox4ΔHMG, or pFlag-Sox4ΔTAD (d). L02 cells were transfected with pFlag2A or pFlag-Sox4. Total mRNA extracts were prepared from the cells. The *p65, p50, IRF3*, and *IRF7* mRNAs expressed in the cells were determined by RT-PCR using the corresponding primers (e). HepG2 cells were co-transfected with pFlag-Sox4 and pGFP-p65, pGFP-p50, pGFP-IRF3, or pGFP-IRF7, and treated with cycloheximide (f). 293 T (left) and HepG2 cells (right) were co-transfected with pFlag-Sox4 and pGFP-p65, pGFP-p50, pGFP-IRF3, or pGFP-IRF7, and treated with MG-132 (g). p-p65, p65, IRF3, IRF7, Sox4, GFP, and GAPDH proteins were detected by Western blot analyses. (h) L02 cells were co-transfected with pMYC-Ub and pFlag-Sox4, pGFP-p65, pGFP-p50, pGFP-IRF3, or pGFP-IRF7, and treated with MG-132. Proteins in cell lysates were detected by Western blot analyses, proteins in supernatant were detected by IP assays or by Western blot analyses. (i) L02 cells were co-transfected with pIFN-β-Luc and pFlag-Sox4, pGFP-p65, pGFP-p50, pGFP-IRF3, or pGFP-IRF7, and infected with SeV. Luciferase activities were measured using a TD-20/20 luminometer. The results are shown as means ± SD (*n* = 3). *P < 0.05

Similar to NF-kB, IRF3, and IRF7 are important regulators of the production of IFNs [43]. Thus, we examined the effect of Sox4 on the regulation of NF-kB, IRF3, and IRF7. Since activation of NF-kB and IRF3/7 leads to nuclear translocations of p65, p50, IRF3, and IRF7, we evaluated the roles of Sox4 in nuclear translocations of the proteins. In the cytoplasm, p65 and IRF7, but not p50 and IRF3, were repressed by Sox4, activated by SeV, and virus-mediated activation was downregulated by Sox4 (Figure 4a, left); and in the nucleus, p65, p50, IRF3, and IRF7 were reduced by Sox4, enhanced by SeV, and virus-mediated enhancement was attenuated by Sox4 (Figure 4a, right). In addition, p65, p50, IRF3, and IRF7 were stimulated by SeV, attenuated by Sox4 (Figure 4b), but enhanced by siR-Sox4 (Figure 4c). These results indicated that Sox4 not only represses nuclear translocation of p65, p50, IRF3, and IRF7, but also inhibits the production of these factors. The levels of p65, p50, IRF3, and IRF7 proteins were not affected by Sox4ΔHMG or Sox4ΔTAD (Figure 4d), suggesting that only wild type Sox4 facilitate the down-regulation of NF-kB and IRF3/7 proteins. However, the levels of *p65, p50, IRF3*, and *IRF7* mRNAs were not affected by Sox4 (Figure 4e), indicating that Sox4 may regulate NF-kB and IRF3/7 at the post-transcriptional level.

We speculated that Sox4 may attenuate NF-kB and IRF3/7 post-translationally by regulating protein stability. There are two major protein degradation systems, the ubiquitin-proteasome pathway and the autophagic-lysosomal pathway [44,45]. Since NF-kB and IRF3/7 are subjected to proteasomal degradation [46], we examined the effects of Sox4 on the stabilities of NF-kB and IRF3/7 by using protein synthesis inhibitor (cycloheximide, CHX) chase assay. p65, p50, IRF3, and IRF7 were reduced by Sox4 in the presence of CHX (figure 4f), indicating that Sox4 facilitates protein degradations. p65, p50, IRF3, and IRF7 were attenuated by Sox4 in the absence of proteasome inhibitor (MG-132), but not affected by Sox4 in the presence of MG-132 (Figure 4g), suggesting that proteasome pathway is involved in Sox4-mediated degradation of p65, p50, IRF3, and IRF7. Moreover, poly-ubiquitinations of p65, p50, IRF3, and IRF7 were detected in the absence of Sox4, but the levels of poly-ubiquitination were significantly enhanced by Sox4 (Figure 4h), demonstrating that Sox4 facilitates ubiquitination-related degradation of NF-kB and IRF3/7.

Since NF-kB and IRF3/7 are essential for activation of IFN, and Sox4 facilitates NF-kB and IRF3/7 degradation, we thus evaluated the effects of NF-kB and IRF3/7 on Sox4-mediated regulation of *IFN-α* promoter. *IFN-β* promoter activity was stimulated by SeV and repressed by Sox4, but Sox4-mediated repression was rescued by p65, p65/p50, IRF7, and IRF3/IRF7 (Figure 4i), suggesting that Sox4-mediated degradation of NF-kB and IRF3/7 plays an important role in the repression of IFN. Taken together, Sox4 facilitates the degradation of NF-kB and IRF3/7, which leads to the repression of IFNs during viral infection.

## Sox4 attenuates MyD88-dependent and -independent pathways and represses MyD88 production

**Figure 5.**
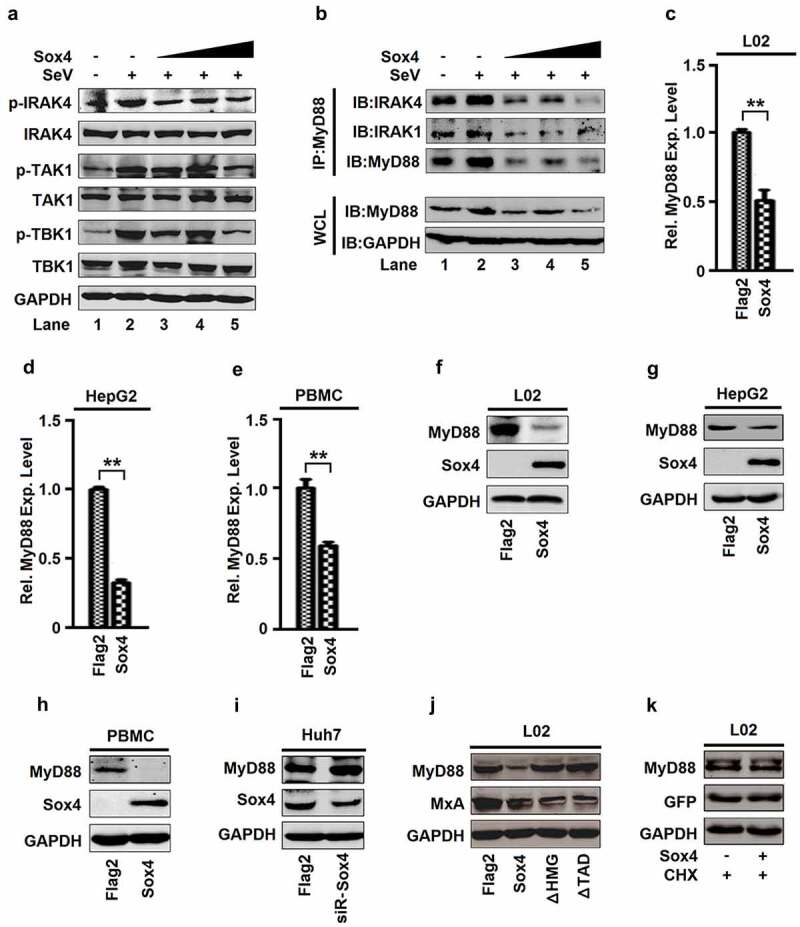
**Sox4 attenuates MyD88-dependent and independent pathways and represses MyD88 expression**. (a and b) L02 cells were transfected with pFlag-Sox4 and infected with SeV. Proteins expressed in the cells were determined by Western blot analyses (a). Whole cell extracts were prepared for Co-IP using antibody to MyD88, and the precipitates were analyzed using antibodies to IRAK4, IRAK1, or MyD88 (B, top). MyD88 and GAPDH proteins in the whole cell lysates (WCLs) were analyzed by Western blots (B, bottom). (c–e) L02 cells (c), HepG2 cells (d), and PBMCs (e) were transfected with pFlag-Sox4 or pFlag2A. *MyD88* mRNAs expressed in the cells was determined by RT-PCR. The results are shown as means ± SD (*n* = 3). **P < 0.05. (f–k) L02 cells (f), HepG2 cells (g), and PBMCs (h) were transfected with pFlag-Sox4 or pFlag2A. Huh7 cells were transfected with siR-Sox4 or siR-NC (i). L02 cells were transfected with pFlag-Sox4, pFlag-Sox4ΔHMG, or pFlag-Sox4ΔTAD (j). L02 cells were transfected with pFlag-Sox4 or pFlag2A and then treated with CHX (k). MyD88, Sox4, GFP, and GAPDH proteins expressed in the cells were detected by Western blot analyses

TLRs induce IFNs by regulating the TLR/MyD88/IRAK4/TAK1 (MyD88-dependent) and TLR/TRIF/TRAF3/TBK1 (MyD88-independent) pathways [47,48]. Here, we evaluated the effects of Sox4 on the regulation of key components in the pathways. The levels of p-IRAK4, p-TAK1, and p-TBK1 were activated by SeV, but repressed by Sox4 (Figure 5a), indicating that Sox4 attenuates both TLR/MyD88/IRAK4/TAK1 and TLR/TRIF/TRAF3/TBK1 pathways in response to viral infections.

Stimulation of TLRs triggers MyD88, which subsequently recruits IRAK4, IRAK1, and TRAF6 to form a complex to initiate signal transduction. We further determined the role of Sox4 in the formation of the MyD88 complex. The interactions of MyD88 with IRAK4 and IRAK1 were enhanced by SeV and repressed by Sox4 (Figure 5b, top), and the production of MyD88 was upregulated by SeV and downregulated by Sox4 (Figure 5b, bottom), suggesting that Sox4 disrupts MyD88/IRAK4/IRAK1 complex formation through repressing MyD88 production. In addition, *MyD88* mRNA (Figure 5c–e) and MyD88 protein (figure 5f–h) were attenuated by Sox4 in L02, HepG2, and PBMC (Figure 5c–h). In addition, MyD88 protein was enhanced by siR-Sox4 in Huh7 cells (Figure 5i). These results demonstrated that Sox4 represses MyD88 expression. MyD88 was repressed by Sox4, but not by Sox4ΔHMG and Sox4ΔTAD (Figure 5j), suggesting that HMG and TAD are required for Sox4-mediated repression of MyD88. Furthermore, MyD88 protein was not affected by Sox4 in the presence of CHX (Figure 5k), indicating that Sox4 represses MyD88 through transcriptional regulation. Taken together, we demonstrated that Sox4 represses the MyD88-dependent pathway by disrupting MyD88/IRAK4/IRAK1 complex formation, and attenuates the MyD88-independent pathway by repressing TBK1 activity.

## Sox4 represses the expression of all TLRs except TLR2

**Figure 6.**
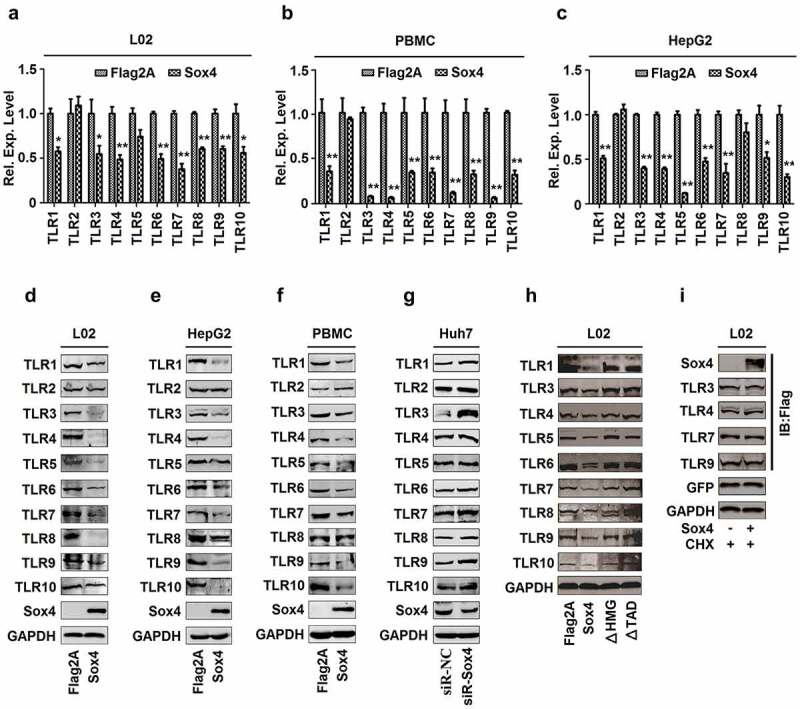
**Sox4 inhibits expression of all TLRs except TLR2**. (a–c) L02 cells (a), PBMCs (b). HepG2 cells (c) were transfected with pFlag2A or pFlag-Sox4. The levels of *TLR1, TLR2, TLR3, TLR4, TLR5, TLR6, TLR7, TLR8, TLR9*, and *TLR10* mRNAs expressed in the cells were determined by RT-PCR. The results are shown as means ± SD (*n* = 3). **P < 0.05. (d–g) L02 cells (d), HepG2 cells (e), and PBMCs (f) were transfected with pFlag2A or pFlag-Sox4. Huh7 cells were transfected with siR-Sox4 or sir-NC (g). TLR1, TLR2, TLR3, TLR4, TLR5, TLR6, TLR7, TLR8, TLR9, and TLR10 proteins produced in the cells were detected by Western blot analyses. (h and i) L02 cells were transfected with pFlag2A, pFlag-Sox4, pFlag-Sox4ΔHMG, or pFlag-Sox4ΔTAD (h). L02 cells were transfected with pFlag2A or pFlag-Sox4 and treated with CHX (i). The protein levels of TLRs produced in the cells were detected by Western blot analyses using corresponding antibodies

Inductions of IFNs are mediated by TLRs and activation of TLRs triggers downstream events of host innate immunity [6,7]. Interestingly, *TLR1, TLR3, TLR4, TLR5, TLR6, TLR7, TLR8, TLR9*, and *TLR10* mRNAs, but not *TLR2*, were repressed by Sox4 in L02 cells and PBMCs (Figure 6a and b), and all *TLRs* mRNAs except *TLR2* and *TLR8* were inhibited by Sox4 in HepG2 cells (Figure 6c). Similarly, TLR1, TLR3, TLR4, TLR5, TLR6, TLR7, TLR8, TLR9, and TLR10 proteins, but not TLR2, were attenuated by Sox4 in L02 and HepG2 cells (Figure 6d and e). The levels of all TLRs except TLR2 and TLR8 were downregulated by Sox4 in PBMCs (figure 6f). All TLRs proteins except TLR2 were upregulated by siR-Sox4 in Huh7 cells (Figure 6g). In addition, all TLRs proteins except TLR2 were repressed by Sox4, but not by Sox4ΔHMG and Sox4ΔTAD (Figure 6h). Furthermore, the levels of TLR3, TLR4, TLR7, and TLR9 proteins were not affected by Sox4 in the presence of CHX (Figure 6i), indicating that Sox4 represses the expressions of TLRs through transcriptional regulation. Taken together, to our surprise, Sox4 plays a general role in repressing transcription of all TLRs except TLR2.

## Sox4 represses TLRs by binding to the promoters resulting in inhibit gene transcription

**Figure 7.**
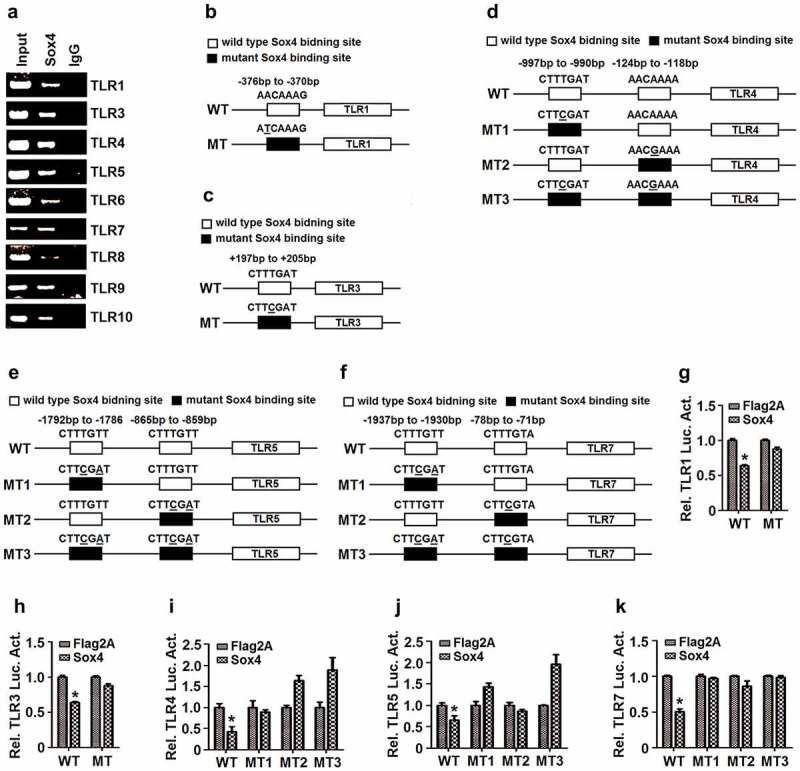
**Sox4 represses TLRs expression by binding to the promoters**. (a) L02 cells were transfected with pFlag-Sox4. The cell extracts were prepared for IP analyses using antibody to Flag, and the precipitated DNA were analyzed by PCR using ChIP primers for *TLR1, TLR3, TLR4, TLR5, TLR6, TLR7, TLR8, TLR9*, or *TLR10*. (b–f) Diagrams of the WT-TLR1 and MT-TLR1 promoters (b); the WT-TLR3 and MT-TLR3 promoters (c); the WT-TLR4, MT1-TLR4, MT2-TLR4, and MT3-TLR4 promoters (d); the WT-TLR5, MT1-TLR5, MT2-TLR5, and MT3-TLR5 promoters (e); and the WT-TLR7, MT1-TLR7, MT2-TLR7, and MT3-TLR7 promoters (f). □ The wild-type of Sox4 binding site on TLR promoter; ■ The mutant Sox4 binding sites on TLR promoter, in which the mutated nucleotides are underlined. (g–k) L02 cells were co-transfected with pFlag-Sox4 along with pWT-TRL1-Luc or pMT-TRL1-Luc (g); with pWT-TRL3-Luc or pMT-TRL3-Luc (h); with pFlag-Sox4 and pWT-TRL4-Luc, pMT1-TLR4-Luc, pMT2-TLR4-Luc, or pMT3-TLR4-Luc (i); with pWT-TRL5-Luc, pMT1-TLR5-Luc, pMT2-TLR5-Luc, or pMT3-TLR5-Luc (j); and with pWT-TRL7-Luc, pMT1-TLR7-Luc, pMT2-TLR7-Luc, or pMT3-TLR7-Luc (k). Luciferase activities in the cell extracts were measured using a TD-20/20 luminometer. The results are shown as means ± SD (*n*= 3). **P< 0.05

Sox4 is a transcription factor and binds preferentially to the AACAAAG/CTTTTGTT motif leading to activate transcription of targeted genes [49]. Interestingly, we revealed that the promoters of all TLRs except TLR2 contain at least one potential Sox4 binding sequence (Table 1). Chip assays showed that Sox4 could bind to the promoters of *TLR1, TLR3, TLR4, TLR5, TLR6, TLR7, TLR8, TLR9*, and *TLR10* (Figure 7a), suggesting that Sox4 directly binds to *TLR* promoters.

**Figure 8.**
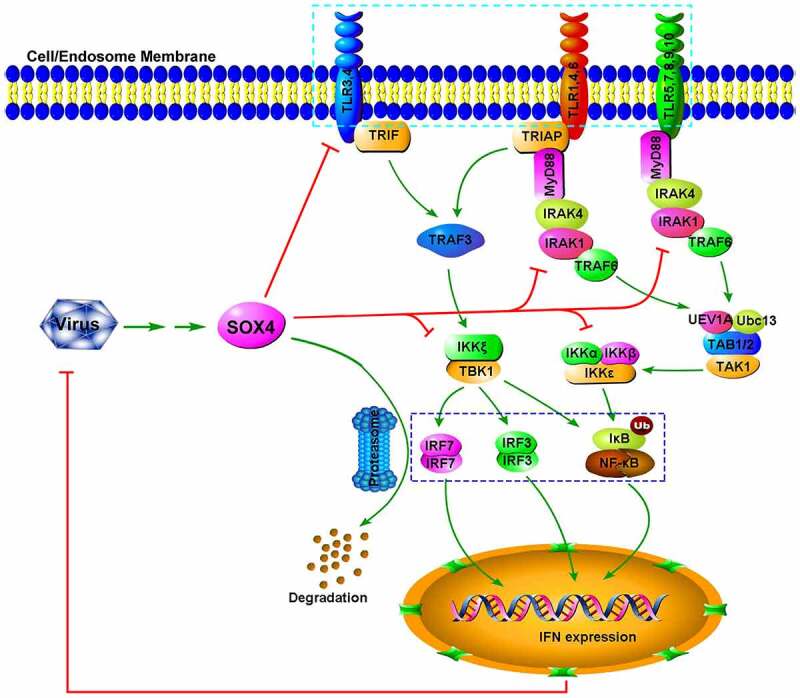
**A proposed mechanism by which Sox4 represses host innate immunity and facilitates pathogen infection**. Sox4 initially is induced during viral infections. Sox4 subsequently represses the expression of *TLRs* by binding to their promoters to inhibit gene expression. Sox4 also attenuates the transcription of *MyD88*, a critical component in the TLR signaling cascades. In addition, Sox4 blocks the TLR/MyD88/IRAK4/TAK1 and TLR/TRIF/TRAF3/TBK1 pathways through repressing the key signaling components. Moreover, Sox4 inhibits NF-kB activity *via* interacting with IKKα/α and further represses NF-kB and IRF3/7 function by inhibiting nuclear translocation and promoting protein degradation. Finally, Sox4 down-regulates the expression of *IFNs* and *ISGs*, which lead to the facilitation of pathogen replication

To confirm the specific binding of Sox4 to *TLR* promoters, we constructed mutant promoters of *TLR1, TLR3, TLR4, TLR5*, and *TLR7* by site-directed mutagenesis of Sox4-binding sequences (Figure 7b–f). The wild-type TLR (WT-TLR) promoters and the mutant TLR (MT-TLR) promoters were inserted into pGL3-Basic to generate corresponding reporter plasmids. L02 cells were co-transfected with pFlag-Sox4 and each of the reporter plasmids expressing WT-TLR-Luc or MT-TLR-Luc. Luciferase assays showed that Sox4 inhibited WT-*TLR1* activity, but not MT-*TLR1* (Figure 7g); repressed WT-*TLR3* function, but not MT-*TLR3* (Figure 7h); down-regulated WT-*TLR4* activity, but not MT1-*TLR4*, and up-regulated MT2-*TLR4* and MT3-*TLR4* (Figure 7i); attenuated WT-*TLR5* activity, but not MT1-*TLR5*, MT2-*TLR5*, or MT3-*TLR5* (Figure 7j); and reduced WT-*TLR7* activity, but not MT1-*TLR7* or MT2-*TLR7*, and enhanced MT3-*TLR7* (Figure 7k). Taken together, we revealed that the binding sequences of Sox4 on *TLR* promoters are required for the regulation of TLRs and demonstrated that Sox4 represses *TLRs* at transcription level through binding and inhibiting *TLR* promoters (Figure 8).

## Discussion

TLRs are mediators critical for the regulation of host immunity to combat pathogen infections [7,50]. However, without the tightly controlled immune responses to TLRs, the host would be subjected to detrimental outcomes, possibly resulting in mortality. Therefore, it is extremely important to balance the positive activation and negative repression of TLR pathways to eliminate viral infection yet avert harmful immunological consequences. Here, we identified that Sox4 acts as a regulator to repress TLR signaling networks and host innate immunity. We initially demonstrated that VSV, HCV, EV71, and IAV activate Sox4, which subsequently facilitates viral replications. Since innate immune responses play important roles in regulating viral replication [51,52], we hypothesized that Sox4 may broadly enhance virus replication through repressing host immunity. Firstly, we showed that Sox4 could repress *IFNs* and *ISGs* expression with stimulation, which suggests that Sox4 plays a role in attenuating *the IFN*-related pathway. Then, we confirmed Sox4 performs this repression through four aspects on the upstream pathway: 1. Sox4 inhibits NF-kB activity by interacting with IKKα/α complex and repressing IκBα phosphorylation, ubiquitination, and degradation. 2. Sox4 facilitates proteasome-mediated ubiquitination-related degradation of NF-kB and IRF3/7 proteins. 3. Sox4 attenuates the phosphorylation of IRAK4, TAK1, and TBK1 to repress the activation of TLR/MyD88/IRAK4/TAK1 and TLR/TRIF/TRAF3/TBK1 pathways. 4. Sox4 downregulates the transcription of all *TLRs* excepting *TLR2* and *MyD88* by directly binding to their promoters.

TLRs initiate IFN/JAK/STAT signaling through regulating TLR/MyD88/IRAK4/TAK1 and TLR/TRIF/TRAF3/TBK1 pathways [6]. Sox4 may act as a master regulator to repress TLR signaling networks and host innate immunity. Although many cellular factors have been reported to suppress TLR signaling *via* different mechanisms [23,26,27], they regulate TLRs by targeting a single molecule or a shared molecule at one stage. Therefore, we at the first time identified a negative regulator involved in controlling TLR signaling pathways at multiple stages. TLRs recognize pathogen-associated molecular patterns (PAMPs) derived from invading pathogens [53]. Most TLRs are localized at the cell surface to recognize bacterial products, whereas TLR3, 7, 8, and 9 are produced in endosomal compartments in response to viral components [54,55]. TLR3 responds to viral dsRNA, TLR7 and TLR8 sense viral ssRNA, whereas TLR9 recognizes viral DNA conformations. We demonstrated that Sox4 represses all TLRs except TLR2, implicating that Sox4 may play general roles in regulating the infections of vast number of pathogens, including viruses, bacteria, fungi, and protozoan, although further studies are needed.

Taken together, we identified a master regulator that hijacks host innate immunity by repressing TLRs signaling networks at multiple stages. The discovery of a master negative regulator and its direct implications in host defenses provides insights into our understanding of pathogen infection and host immunity and provides future possibilities for targeting signaling molecules for new therapeutics in human infections and associated diseases.
